# Draft Genome Sequences of Three Novel Low-Abundance Species Strains Isolated from Kefir Grain

**DOI:** 10.1128/genomeA.00869-17

**Published:** 2017-09-28

**Authors:** Yongkyu Kim, Sonja Blasche, Kiran R. Patil

**Affiliations:** Structural and Computational Biology Unit, European Molecular Biology Laboratory, Heidelberg, Germany

## Abstract

We report here the genome sequences of three novel bacterial species strains—*Bacillus kefirresidentii* Opo, *Rothia kefirresidentii* KRP, and *Streptococcus kefirresidentii* YK—isolated from kefir grains collected in Germany. The draft genomes of these isolates were remarkably dissimilar (average nucleotide identities, 77.80%, 89.01%, and 92.10%, respectively) to those of the previously sequenced strains.

## GENOME ANNOUNCEMENT

Kefir grains are traditionally used as starters for producing kefir—a fermented milk drink. They contain a stable consortium composed of 40 to 50 different species, both prokaryotes and eukaryotes (1). Among the bacterial species, acetic acid bacteria and lactic acid bacteria, including members of the genera *Lactobacillus*, *Lactococcus*, and *Leuconostoc*, are dominant and play important roles in milk fermentation and kefir flavor (2). So far, the functional role of other low-abundance species remains unknown. Here, we report the genome sequences of *Bacillus kefirresidentii* Opo, *Rothia kefirresidentii* KRP, and* Streptococcus kefirresidentii* YK, isolated from kefir grains collected from private sources in Germany.

*B. kefirresidentii* Opo (Internal Stock Code [ISC] 215b) was isolated from ground kefir grains plated in serial dilutions on modified Gifu anaerobic medium (mGAM) agar and grown overnight at 24°C. *R. kefirresidentii* KRP (ISC 156) was isolated from ground kefir grains, plated in serial dilutions on GAM agar and grown for 2 days at 30°C. *S. kefirresidentii* YK (ISC: 384a) was isolated from ground kefir grains, plated in serial dilutions on de Man, Rogosa, and Sharpe agar (MRS)-milk agar (1/1 mix of MRS agar and 3.5% ultrahigh-temperature processing [UHT] milk) and grown for 3 days at 37°C. The isolated clones were suspended in TES buffer (25 mM Tris, 10 mM EDTA, and 10 mM sucrose) and digested with lysozyme for 30 min at 37°C, followed by two bead-beating steps (30 s, 6.5 m/s) with an intermediate break of 1 min. Cells were lysed by the addition of 3% SDS for 5 min at room temperature, followed by proteinase K (0.2 mg/ml final concentration) digestion for 30 min at 37°C. The digested proteins were precipitated for 15 min on ice in 1 M potassium acetate (final concentration) and centrifuged for 15 min at 4°C. The supernatant was purified using standard phenol-chloroform extraction. DNA was precipitated by the addition of 2 volumes of ice-cold isopropanol and washed with 70% ethanol at 4°C.

DNA library creation and sequencing were done at the European Molecular Biology Laboratory genomics core facility (Heidelberg, Germany). It was sequenced on the Illumina HiSeq 2000 platform with 100-bp paired-end reads. The raw reads were assessed for quality-based trimming and filtering by PRINSEQ (3). The qualified read pairs were assembled using SPAdes version 3.10.0 (4). The contigs shorter than 500 bp were discarded.

The total numbers of contigs in the scaffolds are 112, 81, and 131, with largest contig sizes of 774,352 bp, 579,517 bp, and 161,655 bp and *N*_50_ values of 172,674 bp, 94,942 bp, and 92,366 bp, for *B. kefirresidentii* Opo, *R. kefirresidentii* KRP, and *S. kefirresidentii* YK, respectively. The draft genome sizes are 5,057,314 bp (GC content, 45.62%) for *B. kefirresidentii* Opo, 2,522,693 bp (GC content, 58.47%) for *R. kefirresidentii* KRP, and 2,170,993 bp (GC content, 39.31%) for *S. kefirresidentii* YK.

Annotation with the NCBI Prokaryotic Genome Annotation Pipeline identified 5,306, 2,642, and 2,327 protein-coding genes, 77, 51, and 38 tRNAs, and 16, 9, and 5 rRNAs for *B. kefirresidentii* Opo, *R. kefirresidentii* KRP, and *S. kefirresidentii* YK, respectively (5).

### Accession number(s).

This whole-genome shotgun project has been deposited at DDBJ/ENA/GenBank under accession numbers NDFN00000000 for *B. kefirresidentii* Opo, NDFM00000000 for *R. kefirresidentii* KRP, and NGVM00000000 for *S. kefirresidentii* YK.
